# Parasite nucleosomes: Chromatin dynamics rewired

**DOI:** 10.1371/journal.ppat.1013781

**Published:** 2025-12-15

**Authors:** Gauri Deák, Marcus D. Wilson

**Affiliations:** 1 Centre for Cell Biology, University of Edinburgh, Michael Swann Building, Kings Buildings, Mayfield Road, Edinburgh, United Kingdom; 2 Institute of Quantitative Biology, Biochemistry and Biotechnology, University of Edinburgh, Michael Swann Building, Kings Buildings, Mayfield Road, Edinburgh, United Kingdom; Institute of Parasitology, Biology Centre, Czech Academy of Sciences, CZECHIA

## Introduction

Unicellular protist parasites constitute a global threat to human, animal, and plant health. Although the demands of multicellularity are absent in these organisms, they experience rapid environmental changes as they navigate through their life cycles inside a host or vector. A parasitic lifestyle, therefore, necessitates flexible utilisation of resources and phenotypic plasticity; one means to achieve this is through the organisation and regulation of their genome.

**Fig 1:**
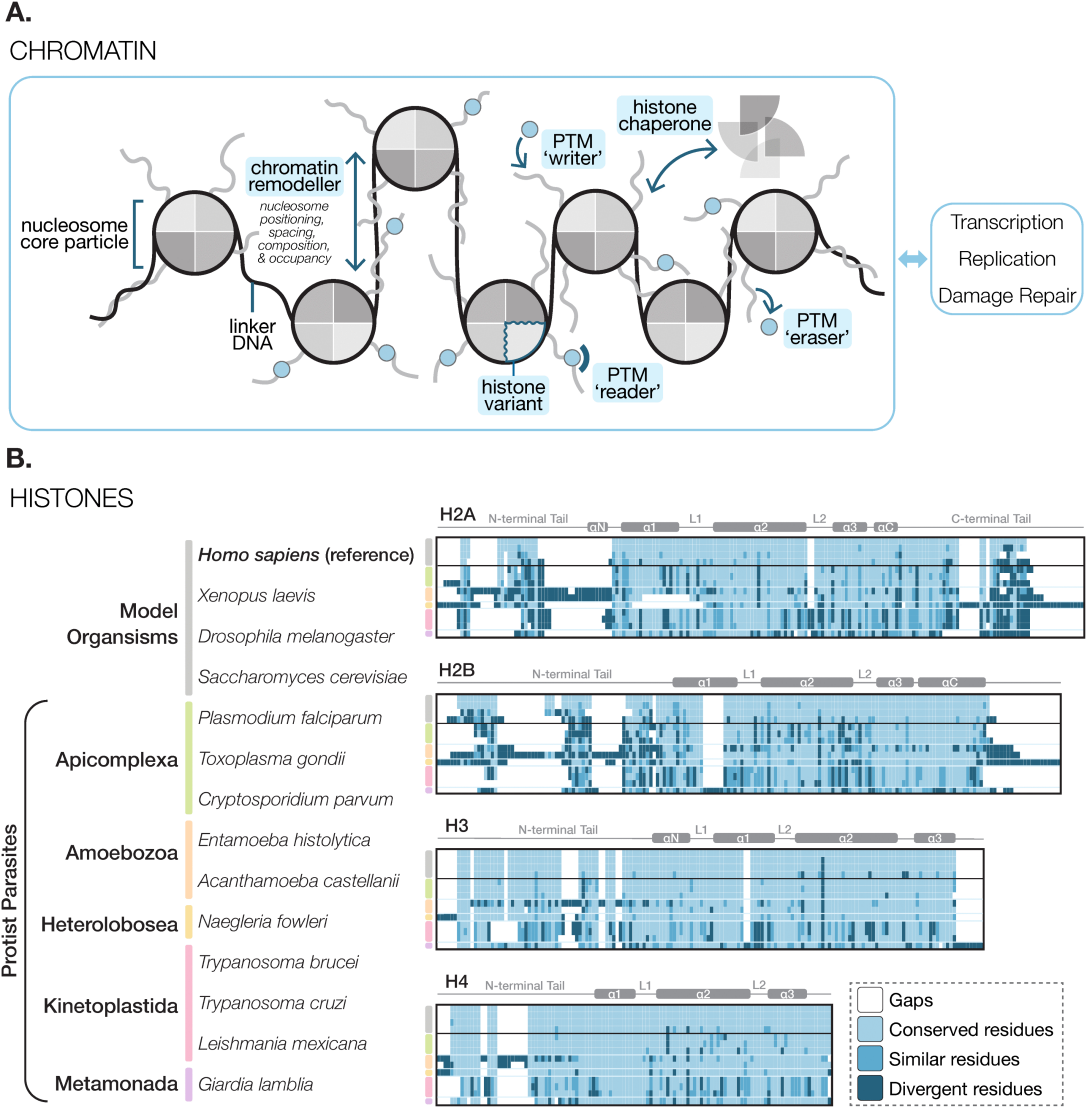
Nucleosomes are the building blocks of chromatin. **A.** Genomic DNA (black) is wrapped around nucleosomes (grey circles), which consist of four histones with a structured core and disordered N- and/or C-terminal tails (grey lines). The nucleosome core particles (NCP) are separated by linker DNA. The properties of histones can be modulated by PTMs (light blue circles; e.g., acetylation, methylation, ubiquitylation, etc.), which are maintained by ‘writers’ that deposit the PTMs, ‘readers’ that bind to the PTMs to recruit downstream factors, and ‘erasers’ that remove the PTMs. Nucleosomes can also undergo dynamic sliding, changes in composition due to replacement of canonical histones with histone variants, and assembly/disassembly by chromatin remodellers or histone chaperones. **B.** Histones are divergent in protist parasites. Protein multiple sequence alignments for each histone (H2A, H2B, H3, and H4) from various model organisms (grey) and protist parasites (coloured by phylum) are shown as heatmaps. The secondary structure of each histone is shown above each alignment (“α” = α-helix, “L” = loop). Conserved residues = residues that are identical to each reference sequence (*Homo sapiens* histones); Similar residues = residues with a PAM250 score > 0.5; Divergent residues = residues with a PAM250 score ≤ 0.5.

In most eukaryotes, genomic DNA is packaged into chromatin, a linear, foldable, and modular polymer. The fundamental unit of chromatin is a nucleosome, which typically consists of two copies of four histone proteins (H2A, H2B, H3, and H4) that assemble into a disk-shaped octamer and wrap ~145 bp DNA [1]. In addition to DNA packaging, nucleosomes modulate access to DNA and serve as a scaffold for protein interactions during essential cellular processes such as DNA transcription, replication, and repair [2] (Fig 1A). Nucleosome function can also be fine-tuned via dynamic histone post-translational modifications (PTMs), histone variants, and active nucleosome remodelling (Fig 1A). However, these processes are often highly divergent in protist parasites.

## Why is chromatin structure in parasites clinically relevant?

The importance of parasite chromatin is underscored by a number of molecular pathways where gene regulation, parasite development, and pathogenicity intersect. For example, recent work revealed that an ISWI-like chromatin remodeller from *Plasmodium falciparum* can slide nucleosomes and likely interacts with histone chaperones and PTM-related proteins, creating permissive or repressive chromatin environments at developmentally-regulated gene promoters [3]. Importantly, inhibition of the remodeller and histone-modifying enzymes impairs parasite gametogenesis, providing a promising avenue for blocking malaria transmission [3,4]. Similarly, ISWI-like remodelling proteins from *Toxoplasma gondii* can influence chromatin accessibility at promoters and are important for parasite viability [5,6].

The role of nucleosomes is also evident in *Trypanosoma brucei,* where most genes are constitutively transcribed, except a silent repertoire of variant surface glycoprotein (VSG) genes, where only one gene is expressed at a time to facilitate evasion of the host adaptive immune response [7]. Nucleosome occupancy and chromatin compaction anti-correlate with VSG expression status [8–10] and histone variants [10], chaperones [11,12], and histone PTM-related proteins [13,14] are crucial for maintaining monoallelic expression of a single VSG gene in the context of specialised 3D chromatin compartments [15]. Antigenic variation occurs through periodic switching of the active VSG gene and involves regulated chromatin re-organisation. Artificial disruption of this system leads to easier parasite clearance and subsequent health in the host [16].

Less is known about chromatin pathways in parasites like *Giardia* and *Entamoeba,* but again, histone PTMs are implicated in developmental changes that are important for parasite transmission [17,18]. These examples highlight that nucleosomes are often at the mechanistic core of gene regulation events and contribute to parasite pathogenicity. Importantly, the essential chromatin pathways in parasites are highly divergent and open therapeutic avenues for designing drugs with minimal off-target effects on the host. Understanding parasitic chromatin will therefore continue to be clinically relevant.

## What makes parasite nucleosomes unusual?

Due to their central, structural function in shaping chromatin architecture and interactions, histones tend to be highly conserved across eukaryotes. However, this is not the case for some parasitic protists (Fig 1B) and is reflected in the properties of their chromatin. For example, chromatin extracted from *Trypanosoma brucei, Trypanosoma cruzi,* and *Entamoeba histolytica* cells is less condensed and more sensitive to nuclease digestion compared to model eukaryotes [19,20]. In part, this may be mediated by divergence (e.g., in trypanosomatids and *Entamoeba*) or even absence (e.g., in *Plasmodium* and *Giardia*) of linker histone H1 [21], and an altered balance of genomic DNA sequence content (e.g., in *Plasmodium)* [22]. However, nucleosomes reconstituted with *Trypanosoma brucei* [23]*, Plasmodium falciparum* [24], and *Giardia lamblia* [25] histones *in vitro* are also more labile when heated or treated with salt. The common denominator across these studies is *decreased* chromatin stability, but it remains unclear why this is the case.

One possibility is that altered chromatin is a function of the local environment. For example, temperature changes between the insect vector and a warm-blooded mammalian host could be associated with global changes that favour chromatin opening. However, in parasites like *T. brucei* and *T. cruzi*, 3D chromatin organisation and bulk nucleosome occupancy are constant across different life cycle stages [26,27], and chromatin from trypanosome mammalian host stages is more compact and less sensitive to nuclease digestion [20,28]. This suggests that the relationship between environmental changes and chromatin compaction is unlikely to be linear and is instead more complex.

Is decondensed chromatin a shared feature of smaller genomes such as those found in protist parasites? Evidence for small genome size coupled to lower nucleosome stability is also apparent from yeasts [29,30], and previous analyses have shown that histone-driven chromatin compaction can co-evolve with genome size to offset discrepancies between genome expansion and limited nuclear volume [31]. It is possible that nucleosome stability could also reflect genome transposon load, transcriptional activity, replication speed, or other chromatin processes. However, systematic comparisons between these parameters and histone evolution are needed to dissect whether the biophysical properties of nucleosomes correlate with chromatin functions across different genome types and scales in parasites.

## What do we know about the structure of parasite nucleosomes so far?

**Fig 2:**
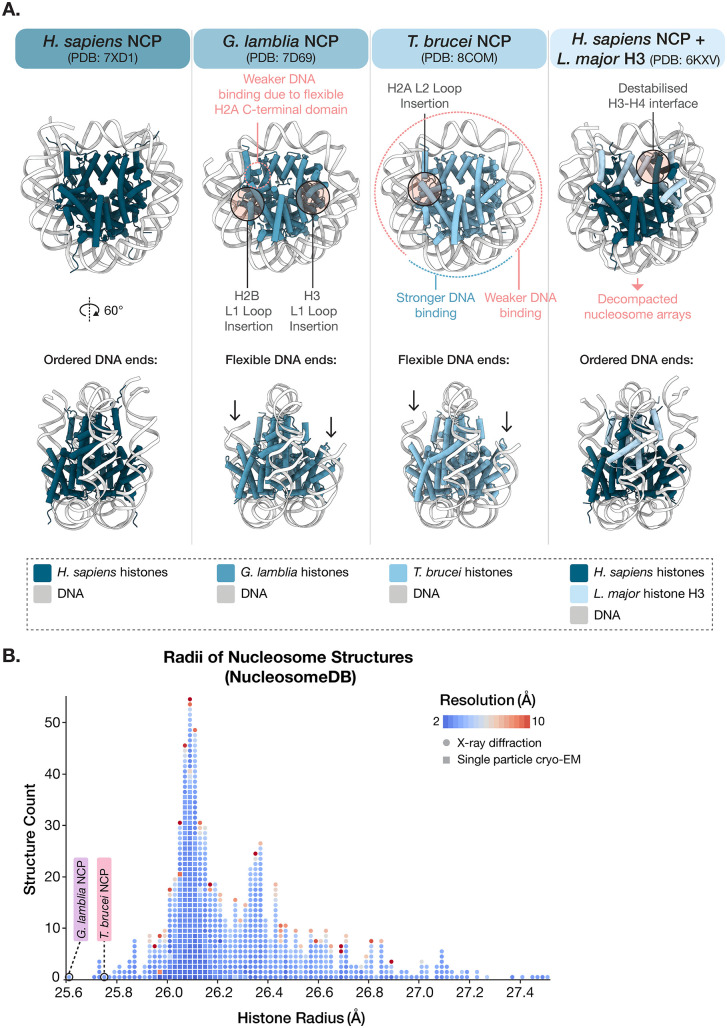
Parasite histones alter nucleosome properties. **A.** Comparison of *H. sapiens*, *G. lamblia, T. brucei,* and chimeric *H. sapiens/L. major* NCP structures; Protein Data Bank (PDB) accession codes: 7XD1 [35], 7D69 [25], 8COM [23], and 6KXV [32] respectively. Top row: Top view of each NCP. Structural differences in loop regions and DNA-binding properties are indicated. Bottom row: Side view of each NCP. The flexible DNA ends of *G. lamblia* and *T. brucei* NCPs are indicated with arrows. **B.** A distribution plot from NucleosomeDB (retrieved in Nov. 2025) [36] showing the radii of the histone protein component of published nucleosome structures. Circles represent structures determined by single particle cryo-EM and squares represent structures solved by X-ray crystallography. The outlier positions of the *G. lamblia* NCP (PDB: 7D69) [25] and *T. brucei* NCP (PDB: 8COM) [23] are indicated. Each structure is coloured based on its resolution.

At the time of writing, the Protein Data Bank contained ~800 published nucleosome structures. Of these, only three comprise parasite histones, namely the structure of the *Giardia lamblia* nucleosome core particle (NCP) [25], the structure of the *Trypanosoma brucei* NCP [23], and the structure of a hybrid NCP composed of *Leishmania major* histone H3 and human histones H2A, H2B, and H4 [32] (Fig 2). These structures revealed that despite considerable histone sequence divergence, the core architecture of the histone fold and how histones pack together in the nucleosome is conserved (Fig 2A). However, sequence divergence at the amino acid level leads to large functional consequences on the biomechanical properties of both *G. lamblia* and *T. brucei* NCPs. Both parasite NCP structures are more oval in shape (Fig 2B) and display weaker DNA binding that leads to displaced DNA ends. They also include structural deviations in histone loop regions, and contain substantial alterations at histone-histone and histone-DNA interfaces (Fig 2A). Even the addition of a single parasite histone in hybrid nucleosomes affects overall nucleosome characteristics [32]. These features provide a biochemical explanation for the reduced stability and loose DNA binding of parasite nucleosomes. They also suggest that parasite nucleosomes are likely to have altered nucleosome (dis)assembly dynamics during DNA-templated events (e.g., the passage of RNA or DNA polymerases). However, the mechanistic effects of altered nucleosome DNA binding and stability remain to be investigated.

## The nucleosome acidic patch: does it support chromatin interactions in parasites?

**Fig 3:**
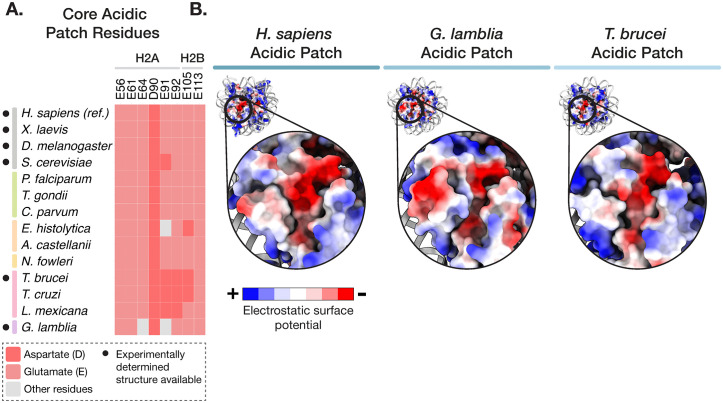
Atypical acidic patches in parasite nucleosomes. **A.** Conservation of acidic patch residues in histones H2A and H2B across model organisms (grey) and protist parasites (coloured by phylum). Residue numbering corresponds to the *H. sapiens* H2A and H2B histone sequences. Species for which experimentally determined structures of nucleosomes are available are indicated with a black circle. **B.** Close-up view of the acidic patch in human, *G. lamblia,* and *T. brucei* NCPs, PDBs: 7XD1 [35], 7D69 [25], and 8COM [23], respectively. DNA is shown as a grey cartoon. Histones are coloured by electrostatic surface potential (red = acidic, blue = basic).

Chromatin proteins interact with nucleosomes by often combining interactions with nucleosomal DNA, the flexible histone tails (which often harbour histone PTMs), and/or the nucleosome histone disk surface. The most common binding hotspot on the disk surface is the acidic patch, which supports varied interactions with chromatin remodelling complexes, histone or DNA modifying enzymes, and signalling factors [2]. The patch comprises eight acidic amino acids in histones H2A and H2B, which are largely conserved in both model organisms and protist parasites (Fig 3A). However, the surrounding amino acids can often be substantially altered and change the local chemical environment of the patch. This is the case for both *T. brucei* and *G. lamblia* nucleosomes, where the acidic patch differs both in shape and surface charge (Fig 3B), preventing interactions with well-characterised binders [23,25]. It is unclear if and how different chromatin proteins have co-evolved to bind a “noncanonical” acidic patch in these parasites.

A recent study revealed that the *T. brucei* histone methyltransferase DOT1A interacts with the nucleosome acidic patch via a flexible loop that is homologous to mammalian/yeast DOT1 enzymes, but divergent at the sequence level [33]. The loops harbours two suspected ‘arginine anchors’, a conserved mechanism of engaging the acidic patch by chromatin proteins [2]. However, further studies are needed to understand the importance of this interaction *in vivo* and to investigate whether the acidic patch is also co-opted by other pathways in *T. brucei*. Furthermore, the histone tails themselves have been described to interact with adjacent nucleosome acidic patches to help aid chromatin compaction in model eukaryotes [1,34]. Whether this is also possible in parasites and could help explain differences in parasite chromatin compaction is a fascinating avenue of future study.

## Future perspectives

The nucleosome serves as a versatile scaffold that compacts DNA and both prevents or facilitates chromatin interactions that are important for parasite development and virulence. The biophysical and structural properties of parasitic nucleosomes differ compared to model eukaryotes, leaving gaps in our present understanding of chromatin biology in these organisms. However, recent advances in genetic manipulation methods, high-throughput–omics, structural approaches, single-molecule technology, and computational predictions hold large promise for answering mechanistic questions on *why* and *how* chromatin dynamics are rewired to facilitate parasite gene regulation. We leave the reader with a few fundamental questions:

- How do the altered DNA-binding properties of parasite nucleosomes affect nucleosome spacing and DNA access by transcription, replication, and repair machinery?
- Does inherent nucleosome instability alter the rate of nucleosome assembly/disassembly dynamics *in vivo*? What are the functional consequences?
- What are the roles of unique histone PTMs, unusual or absent linker histones, and histone variants in parasites?
- How does nucleosome state and chromatin proteins affect interactions with divergent nucleosomes to facilitate parasite life cycle progression and antigenic variation in the host?
- How do nucleosomes across divergent parasite species differ in composition and function? Are there any patterns or histone motifs that can explain the balance between structural constraints on nucleosome architecture and nucleosome plasticity?
